# Evaluating the long-term cost-effectiveness of the COBRA-BPS programme in Pakistan

**DOI:** 10.1136/bmjph-2025-002981

**Published:** 2025-12-03

**Authors:** Carlos Chivardi, Zainab Samad, Tao Chen, Simon Mark Walker

**Affiliations:** 1 Centre for Health Economics, University of York, York, UK; 2 Medicine, Aga Khan University, Karachi, Pakistan; 3 Duke University, Durham, North Carolina, USA; 4 Department of Clinical Sciences, Liverpool School of Tropical Medicine, Liverpool, England, UK

**Keywords:** economics, Community Health, Cardiovascular Diseases

## Abstract

**Introduction:**

The Control of Blood Pressure and Risk Attenuation-Bangladesh, Pakistan, Sri Lanka (COBRA-BPS) programme is a community-based initiative for managing hypertension in rural South Asia. Previous analyses found the intervention more effective but too costly to be considered cost-effective, using a gross domestic product-based threshold cost-effectiveness threshold that overlooked Pakistan’s constrained healthcare resources. Additionally, key benefits, such as avoided cardiovascular events and associated cost savings, were not considered. This study evaluates the long-term cost-effectiveness of the COBRA-BPS programme compared with the standard of care (SoC) in Pakistan.

**Methods:**

A Markov model was used to estimate long-term costs and health outcomes, measured in life years and disability-adjusted life years (DALYs). Cost-effectiveness was assessed using incremental cost-effectiveness ratios (ICERs) and net monetary benefit statistics. Thresholds of US$183 (based on Pakistan’s marginal productivity of public health expenditure), US$500 and US$1000 per DALY averted were applied. Sensitivity analyses were conducted to assess robustness.

**Results:**

Based on the pooled results, the COBRA-BPS programme incurred higher costs than SoC, with an incremental cost of US$105 and an incremental gain of 0.41 DALYs averted over individuals’ lifetimes, resulting in an ICER of US$252 per DALY averted.

**Conclusion:**

COBRA-BPS effectively reduces cardiovascular events but has marginal cost-effectiveness when evaluated against the US$183 per DALY threshold. However, it becomes cost-effective at higher thresholds. Compared with previous analyses, our study found a significantly lower cost per DALY averted, driven by substantial downstream cost savings from avoided cardiovascular events.

**WHAT IS ALREADY KNOWN ON THIS TOPIC**

- Previous evaluations of the Control of Blood Pressure and Risk Attenuation-Bangladesh, Pakistan, Sri Lanka (COBRA-BPS) programme demonstrated its effectiveness in reducing systolic blood pressure in rural South Asian populations, including Pakistan. However, cost-effectiveness analyses relied on thresholds based on gross domestic product, which do not reflect the constrained resources of Pakistan’s public healthcare system. Additionally, prior analyses did not account for the potential long-term cost savings from avoided cardiovascular events, leading to an incomplete assessment of the programme’s true value.

**WHAT THIS STUDY ADDS**

- This study provides a long-term cost-effectiveness evaluation of the COBRA-BPS programme, incorporating downstream cost savings from prevented cardiovascular events. By using a more realistic, productivity-based cost-effectiveness threshold of US$183 per DALY averted, alongside US$500 and US$1000 thresholds, the study demonstrates that while COBRA-BPS is not cost-effective at the lowest threshold, it becomes cost-effective at higher thresholds, reflecting increased willingness to pay. This analysis also highlights significantly lower incremental cost-effectiveness ratios compared with previous studies.

**HOW THIS STUDY MIGHT AFFECT RESEARCH, PRACTICE OR POLICY**

- This study offers policymakers a more realistic understanding of the economic value of COBRA-BPS in resource-constrained settings. It underscores the importance of adopting context-specific cost-effectiveness thresholds and considering long-term health gains and cost savings. The findings can guide healthcare investment decisions in Pakistan and other low- and middle-income countries facing similar resource limitations.

## Introduction

 Hypertension, often termed a ‘silent killer’, is one of the most significant public health concerns worldwide. The burden of hypertension is especially high in South Asia, a region characterised by a high prevalence of risk factors, including poor diet, lack of physical activity and socioeconomic challenges.^1 2^ Hypertension is a key risk factor contributing to the onset of severe cardiovascular diseases (CVDs) such as stroke, myocardial infarction (MI) and heart failure (HF). These conditions are among the leading causes of mortality and morbidity in the region. According to the Global Burden of Disease (GBD) study, stroke alone was the third leading cause of death globally in 2021, resulting in 7.3 million deaths and contributing to 160.5 million disability-adjusted life years (DALYs). Overall, CVDs remained the top cause of global mortality in 2022, with high systolic blood pressure (SBP) identified as a major contributing factor to CVD DALYs, quantified at 2564.9 per 100 000 people worldwide.^3^,^5^

The Control of Blood Pressure and Risk Attenuation-Bangladesh, Pakistan, Sri Lanka (COBRA-BPS) programme, a multi-component hypertension management programme, was developed to reduce blood pressure and alleviate the disease burden of hypertension.^6^ The components in the programme included (1) home health education by government community health workers (CHWs), (2) blood pressure monitoring and referral, (3) training of public and private providers in management of hypertension, (4) designated hypertension triage counters and care coordinators in government clinics and (5) a financing model to compensate for additional services equivalent to 20% of the salary of CHWs. The programme, implemented in rural communities across Bangladesh, Pakistan and Sri Lanka, aimed to reduce blood pressure through a combination of community-based interventions and tailored healthcare strategies. These interventions were designed to be culturally sensitive and require minimal resourcing, with the aim of ensuring sustainability in resource-constrained settings. The programme included strategies such as lifestyle modifications, CHW engagement and low-cost antihypertensive medications, all of which aim to achieve a sustained reduction in blood pressure among participants.

A large randomised controlled trial of the COBRA-BPS programme demonstrated its effectiveness in lowering SBP across various demographic groups. The primary effectiveness analysis found that SBP in rural communities decreased by a mean of 4.39 mm Hg (95% CI 7.84 to 0.94) in Bangladesh, 4.99 mm Hg (95% CI 9.63 to 0.35) in Pakistan and 6.22 mm Hg (95% CI 8.98 to 3.45) in Sri Lanka.^7^ Despite the evident clinical benefits of COBRA-BPS in reducing SBP, its cost-effectiveness, particularly in the long term, remains uncertain. A previous economic evaluation, using a lifetime horizon, found COBRA-BPS to be cost-effective when assessed against a high three times the gross domestic product (GDP)-based cost-effectiveness threshold of US$5090 for Bangladesh, US$4450 for Pakistan and US$12 310 for Sri Lanka per DALY averted (three times the GDP per capita) with ICERs estimated at US$3430, US$2270 and US$4080, respectively.^7^ However, such GDP-based thresholds have been criticised as aspirational and not representative of the true opportunity costs resulting from imposing costs on resource-limited healthcare systems like Pakistan and therefore their use can potentially damage public health.^8 9^ If a productivity-based threshold of US$183 per DALY averted, which is more representative of how much health can be generated at the margin by public expenditure in Pakistan’s healthcare system, COBRA-BPS would not be cost-effective based on the previous cost-effectiveness analysis.^10^ This raises important questions about whether COBRA-BPS should be prioritised for the use of the limited public healthcare resources in Pakistan. If the resources devoted to COBRA-BPS could generate more health benefits if allocated elsewhere, then the programme’s implementation could lead to a decrease in overall population health. The prior evaluation was also limited, as it did not explicitly account for downstream cost savings from avoided cardiovascular events (eg, acute and post-event care), potentially understating cost offsets. Our analysis addresses this by explicitly modelling cardiovascular events and associated costs, capturing potential downstream savings that were not considered previously.

This study aims to conduct a cost-effectiveness evaluation of the COBRA-BPS programme in Pakistan from the perspective of the healthcare system, focusing on its impact on individuals with hypertension over their lifetimes. We will assess not only the programme’s ability to reduce the incidence of major cardiovascular events such as MI, stroke and HF but also the associated costs of each event type and the overall lifetime cost-effectiveness of the programme. Our analysis will judge the outcomes against alternative cost-effectiveness thresholds, including a marginal productivity-based threshold which more realistically represents the opportunity costs of public health expenditure in Pakistan.^11^

## Methods

### Overview

This study evaluated the cost-effectiveness of the COBRA-BPS programme compared with standard of care (SoC) using a decision-analytic Markov model. The model simulated the natural progression of hypertension and cardiovascular events in a hypothetical cohort of Pakistani adults with hypertension but no prior CVD, based on Framingham risk equations. A lifetime horizon was adopted to reflect the chronic nature of hypertension and its long-term complications. The model was populated with data from the COBRA-BPS study, the GBD study and targeted literature reviews. It estimated healthcare costs from a healthcare (public payer) perspective and health outcomes, measured in life years and DALYs. Incremental cost-effectiveness ratios (ICERs) and net monetary benefit statistics were calculated, comparing the health gains from COBRA-BPS with those achievable through alternative resource uses. Cost-effectiveness thresholds of US$183 (based on Pakistan’s marginal productivity of public health expenditure), US$500 and US$1000 per DALY averted were applied. The model was calibrated to align with outcomes observed in the COBRA-BPS trial, enhancing the robustness of the result. The base-case economic evaluation adopts a lifetime horizon to reflect the chronic nature of hypertension and downstream CVD complications. For interpretability, we additionally report cumulative incidence of stroke, MI and HF at 5 and 10 years (corresponding to short- and medium-term programme and budget cycles) and at 30 years, which for our cohort approximates a near-lifetime follow-up and captures the vast majority of modelled events.

### Model structure

We developed a decision analytic model based on a previous model,^12^ which was designed to capture hypertension and the subsequent development of cardiovascular events, integrating a natural disease progression pathway with potential intervention effects. Health states included hypertension, stroke, poststroke, coronary heart disease (CHD), post-CHD, HF, post-HF and death. These states were chosen to represent the potential outcomes for individuals with hypertension, allowing for transitions between them. Individuals entered the model in the hypertension state, where they would remain until they experienced a cardiovascular event (eg, stroke, CHD and HF) or death in each cycle. If no transition occurs, the individual remains in the state of hypertension, accruing routine hypertension-care costs and utility for that cycle. Post-event states (eg, poststroke, post-CHD and post-HF) were included to reflect the increased risk of subsequent events and mortality following a primary cardiovascular event. Transitions between states were based on probabilities that are adjusted for age and tailored to the progression patterns observed within the targeted health conditions (further details in online supplemental table A1). The model cycle length was set to 1 year to accommodate annual updates in health risk assessments, and a half-cycle correction was applied to enhance the accuracy of the model’s projections. The model diagram could be found in the online supplemental material figure A1.

### Patient population

The patient population was based on the COBRA-BPS trial patient population. Of 11 510 people who were screened for the trial, 2645 (23.0%) were enrolled from April 2016 through February 2017. The mean (±SD) age of the participants was 58.8±11.5 years; 64.3% were women, 25.8% had diabetes and 41.9% had chronic kidney disease (CKD). Blood pressure was uncontrolled by 69.6% of the participants and very poorly controlled by 29.6%. More details can be found elsewhere.^13^

### Risk equations

Risk of primary cardiovascular events was based on the Framingham risk equations, which are well-established models for predicting the likelihood of cardiovascular events based on multiple risk factors, including diabetes, smoking status, cholesterol, SBP, etc. Full details of the risk equations are provided elsewhere.^14^,^16^

The main treatment effect was a mean reduction of 4.99 mm Hg in the COBRA-BPS intervention group, which reduced the probability of cardiovascular events in the intervention group.^13^ Other model inputs were obtained from the GBD study and targeted literature reviews. All-cause mortality rates were age- and sex-specific, derived from national life tables^17^ and adjusted using relative risk factors from the literature^18^,^21^ to reflect the increased mortality risk associated with cardiovascular events. See more details in the online supplemental tables A2-A5. Calibration was undertaken only to align absolute risk to the mortality observed in the COBRA-BPS trial. We applied multiplicative scaling factors to the relevant baseline hazards (intercepts) and iteratively searched for the values that minimised the least-squares distance between model-predicted and observed cumulative all-cause mortality at prespecified time points (trial follow-up). Covariate coefficients and relative effects were not re-estimated.

### Cost estimation

Costs included in the model include those associated with intervention, hypertension and cardiovascular events. Costs were obtained from the literature (see online supplemental table A6 for complete documentation). All costs were considered from a healthcare perspective and included direct medical expenses, such as medication, hospitalisations, outpatient visits and management of post-event conditions. We do not include non-medical or indirect costs borne by patients/families, such as transportation, meals, time costs, informal care, etc. The cost of the COBRA-BPS intervention was directly sourced from the COBRA-BPS study, which provided data on the intervention expenses, including the costs of training, personnel, medications and other related healthcare services.^22 23^ This study offered a detailed breakdown of the costs associated with implementing the intervention in a real-world setting, ensuring that our model accurately reflects the economic burden of scaling up the COBRA-BPS programme in a similar context.

Additional costs for managing hypertension and cardiovascular events (eg, stroke, HF and MI) were derived from various published studies (ref) that included cost data specific to the Pakistani healthcare system. These studies provided cost estimates based on local healthcare practices and pricing, thus ensuring relevance to our study population. Costs were inflated where appropriate to 2023 values using the Consumer Price Index for Pakistan.^24^ Full details of costs are provided in the online supplemental table A6.

### DALYs estimation

The calculation of DALYs in this study involved the combination of years of life lost (YLL) due to premature mortality and years lived with disability (YLD) due to non-fatal health outcomes. DALYs were estimated using life tables from Pakistan for the year 2022, obtained from the GBD) study.^25^

For calculating YLL, we used the standard life expectancy at each age, as outlined in the 2022 life tables, to determine the number of years lost due to premature death.^26^ The YLD component was calculated by multiplying the prevalence of each health state (eg, stroke, HF and post-MI) by the corresponding disability weight, also sourced from the GBD study. These disability weights represent the severity of health loss associated with each condition on a scale from 0 (perfect health) to 1 (equivalent to death) and were applied to account for the reduced quality of life experienced by individuals living with these conditions.

By combining YLL and YLD, we derived the total DALYs over the lifetime of the hypothetical cohort. This allowed us to quantify the overall burden of hypertension and related CVDs in terms of both premature mortality and morbidity, thus providing a measure of health losses for each treatment considered.

### Cost-effectiveness assessment

We followed the Consolidated Health Economic Evaluation Reporting Standards.^27^ See more details in online supplemental table A7. The economic evaluation focused on estimating the healthcare costs and health outcomes associated with the COBRA-BPS programme versus SoC. Health outcomes were measured in terms of life years and DALYs averted, and costs were measured from a healthcare perspective. The costs and health outcomes were discounted at 3% per annum in line with the international reference case for economic evaluation.^28^ Cost-effectiveness was presented using ICERs and net monetary benefits, using a cost-effectiveness threshold of US$183, reflecting the marginal productivity of the Pakistani healthcare system of US$500 and US$1000 per DALY averted.

### Probabilistic sensitivity analysis (PSA)

PSA was conducted to quantify the impact of parameter uncertainty on the cost-effectiveness results. For each of the 10 000 iterations, we jointly sampled values for all model parameters from their prespecified uncertainty distributions (see online supplemental table A2). The simulated population reflected the baseline characteristics of the trial cohort, which were used to parameterise the Framingham risk equations in the model. These baseline characteristics were fixed across PSA iterations, ensuring a consistent population across simulations so that the PSA reflected uncertainty and not variability. Uncertainty was captured through the sampling of model parameters, most notably the coefficients of the Framingham risk equations and other inputs listed in online supplemental tables A3–A5. For each outcome (cost, QALY, incremental cost and incremental QALY), we report the equal-tailed 95% interval given by the 2.5th and 97.5th percentiles of the empirical PSA distribution. This approach generates a distribution of potential outcomes, allowing us to estimate the probability of COBRA-BPS being cost-effective at different cost-effectiveness thresholds.

### Patient and public involvement

None.

## Results

The cumulative incidence rates of stroke, HF and MI over 5, 10 and 30 years for males and females under the SoC and the COBRA-BPS intervention are shown in table 1. For both men and women, the overall trend shows that the incidence of these conditions increases over time. However, the COBRA-BPS treatment consistently shows slightly lower incidence rates compared with the SoC. This trend is observed across all conditions and for both genders. Across endpoints and time horizons, the median decrease in cumulative incidence of COBRA compared with standard care is 3.9%, ranging from 0.1% for the 30-year cumulative HF incidence to 8.8% for the 5-year cumulative stroke incidence.

**Table 1:** Incidence of cardiovascular events by 5, 10 and 30 years

	Stroke		Heart failure		Myocardial infarction	
Incidence	SoC	COBRA	SoC	COBRA	SoC	COBRA
Males						
5 years	0.026	0.025	0.005	0.005	0.103	0.098
10 years	0.048	0.045	0.010	0.010	0.187	0.179
30 years	0.093	0.088	0.020	0.020	0.359	0.348
Females						
5 years	0.015	0.014	0.003	0.003	0.104	0.099
10 years	0.028	0.026	0.006	0.006	0.190	0.182
30 years	0.057	0.052	0.013	0.013	0.379	0.368

COBRA, Control of Blood Pressure and Risk Attenuation; SoC, standard of care.

The total cost of being in different health states, hypertension, HF, MI and stroke, broken down by sex and treatment arm (COBRA-BPS vs SoC), is shown in table 2. The results show that the costs associated with being in the hypertension state (for both men and women) are higher for those receiving COBRA-BPS, likely reflecting a longer time spent in that state due to the lower incidence of events like MI, stroke and HF and therefore progression out of the hypertension state. In comparison, the costs associated with HF, MI and stroke are slightly lower with COBRA-BPS, indicating fewer occurrences of these more severe events in that group.

**Table 2:** Total cost by treatment and sex

Sex	Treatment	Heart failure (US$)	Hypertension (US$)	Myocardial infarction (US$)	Stroke (US$)	Total (US$)
Female	COBRA	390	5107	1941	137	7575
Female	SoC	401	4779	1886	145	7211
Male	COBRA	388	4767	1761	180	7096
Male	SoC	399	4459	1812	188	6858

Total costs (US$) associated with major cardiovascular events by sex and treatment strategy. These estimates illustrate the distribution of costs across events and highlight differences between COBRA and SoC arms.

COBRA, Control of Blood Pressure and Risk Attenuation; SoC, standard of care.

The cost-effectiveness of the SoC versus the COBRA programme for both males and females, focusing on costs and DALYs, is compared in table 3. For both genders, the COBRA-BPS programme results in slightly higher overall costs and lower DALYs compared with the SoC. The ICER indicates that the COBRA-BPS provides modest improvements in health outcomes (measured by DALYs averted) for a low additional cost. Additionally, individuals under the COBRA-BPS tend to have slightly more years alive compared with the SoC, with an increase of 0.17 years for males and 0.18 years for females.

**Table 3:** Results of the cost-effectiveness analysis

Strategy	Cost (US$)	DALYs	Incremental cost (US$)	Incremental DALYs	ICER	Years alive
Males						
Standard of care	5987	13.09	NA	NA	NA	20.33
CrI 95%	(4966 to 7172)	(9.70 to 16.65)				(18.87 to 21.74)
COBRA programme	6088	12.66	101	0.43	US$234.34	20.50
CrI 95%	(5094 to 7258)	(9.36 to 16.18)	(55 to 146)	(0.34 to 0.52)		(19.08 to 21.88)
Females						
Standard of care	6152	11.55	NA	NA	NA	2
CrI 95%	(5078 to 7413)	(8.50 to 15.21)				(19.90 to 23.03)
COBRA programme	6260	11.14	108	0.41	US$263.41	2
CrI 95%	(5198 to 7467)	(8.16 to 14.80)	(58 to 155)	(0.30 to 0.52)		(20.10 to 23.17)
Pooled results						
Standard of care	6078	12.11				21.11
CrI 95%	(5041 to 7184)	(8.87 to 15.60)				(19.45 to 22.57)
COBRA programme	6183	11.7	105	0.416	US$252.40	21.3
CrI 95%	(5174 to 7265)	(8.52 to 15.20)	(60.7 to 150.7)	(0.32 to 0.50)		(19.66 to 22.71)

Mean discounted costs, DALYs, ICERs and years alive for males, females and pooled populations under standard of care and the COBRA-Bangladesh, Pakistan, Srilanka programme. Values in brackets indicate the 95% CrI, representing the range within which the true value is expected to lie with 95% probability, based on the probabilistic sensitivity analysis.

COBRA, Control of Blood Pressure and Risk Attenuation; CrI, credible intervals; DALYs, disability-adjusted life years; ICERs, incremental cost-effectiveness ratios.

When pooling results across sexes, the COBRA-BPS programme similarly shows higher average costs and fewer DALYs than the standard of care. The incremental analysis yields an average additional cost of US$105 and 0.416 DALYs averted, with a corresponding ICER of US$252.40. On average, individuals in the COBRA-BPS group also live longer, with an increase of 0.19 years alive compared with those in the SoC.

**Figure 1:**
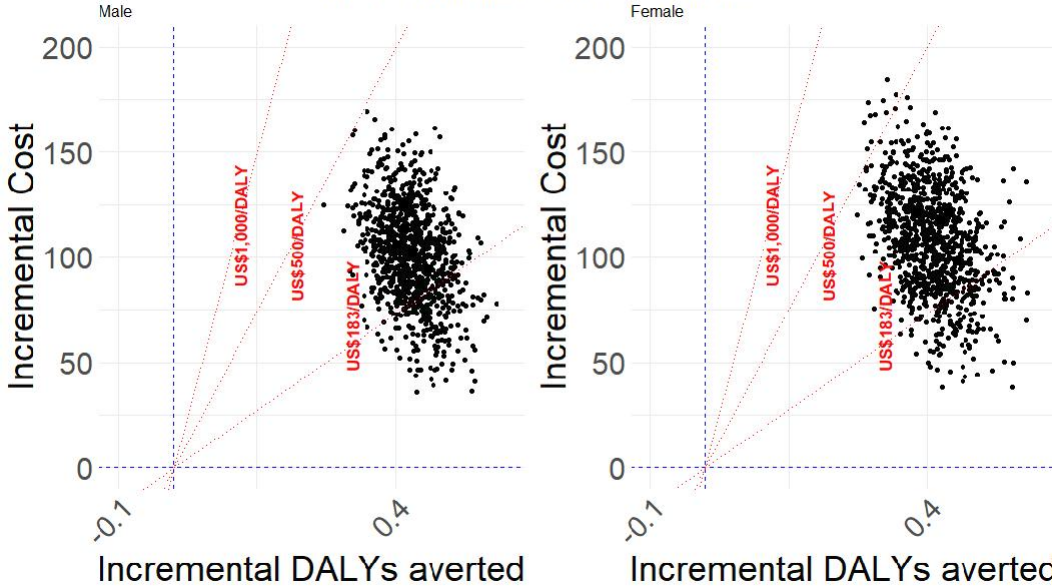
Incremental cost-effectiveness plane. Note: Each dot is a probabilistic sensitivity analysis simulation. The x-axis shows incremental DALYs averted (right=more health) and the y-axis shows incremental cost (up=higher cost). Red dotted lines mark cost-effectiveness thresholds of US$183, US$500 and US$1000 per DALY; points below a line indicate an ICER below that threshold (ie, cost-effective). Blue dashed lines denote the zero axes. DALYs, disability-adjusted life years; ICER, incremental cost-effectiveness ratio.

The results of the PSA, showing the uncertainty in the cost-effectiveness of COBRA-BPS for males (left plot) and females (right plot), are illustrated in figure 1. Each point represents a potential outcome based on varying input parameters, highlighting the spread of incremental costs and incremental DALYs averted under different scenarios. The probability of COBRA-BPS being cost-effective, defined as having an ICER below the threshold of 183, is approximately 20% for males and about 15% for females. For the US$500 threshold, the probability increases to 100% for males and 98% for females, and for the US$1000 threshold, it reaches 100% for both males and females.

## Discussion

The results of our analysis provide a detailed evaluation of the cost-effectiveness of the COBRA-BPS intervention compared with the SoC for both males and females over their lifetime. The findings consistently show that the COBRA-BPS programme reduces the incidence of major cardiovascular events such as stroke, HF and MI compared with the standard care across 5, 10 and 30 years. For example, the COBRA-BPS programme led to a reduction in stroke incidence from 2.68% to 2.52% in males and from 1.57% to 1.43% in females over a 5-year period; at 10 years, stroke incidence declined from 4.86% to 4.58% in males and from 2.87% to 2.63% in females; at 30 years, stroke incidence declined from 9.34% to 8.88% in males and from 5.74% to 5.30% in females. Similarly, the intervention was associated with lower cumulative incidences of HF and MI, demonstrating its potential effectiveness in mitigating the progression of CVD. These findings provide insights into the effects of the intervention on CVD incidence and healthcare costs over a lifetime horizon. The reduction in SBP among participants not only points to the effectiveness of the programme in immediate terms but also suggests significant long-term benefits. By projecting these outcomes over the lifetime of participants, our analysis indicates that sustained blood pressure management can decrease the cumulative incidence of major cardiovascular events, notably stroke, MI and HF.

While the COBRA-BPS programme did achieve health benefits in terms of reduced incidence of cardiovascular events and lower DALYs compared with standard care, these benefits come at a higher cost of healthcare. Although the programme results in slightly higher overall costs for both males (an incremental increase of US$101) and females (an incremental increase of US$108) compared with standard care, it provides a modest reduction in DALYs, with 0.43 DALYs averted for males and 0.41 DALYs averted for females. The ICER is US$234.34 per DALY averted for males and US$263.41 for females. These ICERs are significantly lower than those reported in previous analyses, where the ICERs were US$2270 for Pakistan. However, the ICER is higher than the publicly funded productivity-based threshold of US$183 per DALY averted, which more accurately reflects the economic realities of Pakistan’s health system. This threshold is significantly lower than those typically based on GDP per capita, suggesting a more stringent criterion for evaluating health investments in the context of Pakistan’s limited healthcare budget. The application of a productivity-based threshold reflects the resource constraints within publicly funded health programmes in Pakistan and raises important questions about the allocation of scarce resources. However, this productivity-based threshold is based on public health expenditure only and given the mixed healthcare system of Pakistan, where significant out-of-pocket expenditure exists for most healthcare, it may represent an underestimate of the true relevant cost-effectiveness threshold. Moreover, its use challenges the notion of applying generic cost-effectiveness thresholds, such as those based on multiples of GDP, which may not adequately reflect the opportunity costs and the marginal utility of health spending in low-resource settings. An important limitation of our study is that we report results from a health-system perspective only; incorporating a societal perspective would require reliable local data on productivity, informal care and patient costs and should be a priority for future research.

This study has several strengths. This lifetime analysis offers a detailed look at the long-term effects of the COBRA-BPS programme on hypertension management in Pakistan. Employing a Markov model, the research captures the chronic nature of hypertension and simulates the progression of related cardiovascular events over an extended period. This methodological approach allows for an assessment not only of immediate health outcomes but also of long-term impacts on health and economic costs. The model incorporates detailed clinical data from the COBRA-BPS study and robust cost estimates from local sources, ensuring that the results are relevant to the Pakistani context. However, the study’s applicability to other settings may be limited due to its specific focus on Pakistan, which could affect the generalisability of the findings. Our model population, rural hypertensive adults included in the original COBRA-BPS study, may differ in important respects from the Pakistani hypertensive adult population and the historical Framingham cohorts from which the risk equations were derived. Compared with a recent urban hypertension study in Pakistan,^29^ participants in COBRA-BPS were older on average (mean age 57 years vs mid-40s), and women were over-represented (61% in COBRA vs 50%), and smoking was lower (13% compared with 14%). These differences indicate that the rural population from the study may not be reflective of all hypertensive patients in Pakistan, particularly those in urban areas. Relative to the original Framingham derivation cohorts (North American, predominantly white populations with mean ages in the mid-40s and lower prevalence of uncontrolled hypertension and diabetes), the COBRA population was older, more often female and ethnically South Asian, with higher baseline cardiovascular risk and distinct clinical management patterns. Such demographic and risk-factor differences raise questions about the direct transportability of Framingham-based equations, which were not designed for South Asian populations with these characteristics.

We sought to mitigate these concerns by calibrating modelled risks to trial-consistent outcomes and by exploring sensitivity analyses. Nevertheless, some residual misprediction is possible, and these differences in population characteristics and ethnicity should be considered when interpreting our findings. Nonetheless, some residual miscalibration is possible, and results should be interpreted with caution. The Markov model, while robust, relies on several assumptions regarding disease progression, treatment effects and healthcare utilisation, introducing a level of uncertainty that could affect the predictions of long-term cost-effectiveness. A limitation is that we did not explicitly model the risk of CKD, of which hypertension is a well-established risk factor. The exclusion of CKD-related morbidity and associated treatment costs, such as dialysis, may lead to a conservative estimate of the cost-effectiveness of COBRA-BPS. Future research should explore a more comprehensive modelling approach that incorporates CKD progression and its economic burden. To address the inherent uncertainties and assumptions in the Markov model regarding disease progression, treatment effects and healthcare utilisation, the study has effectively employed PSA. The PSA enhances the robustness of the study by allowing for the simultaneous variation of all model parameters according to their probability distributions. This approach not only captures the range of potential outcomes but also provides a complete assessment of how sensitive the results are to changes in key parameters. By doing so, it quantifies the degree of uncertainty associated with the model’s predictions and demonstrates the conditions under which the COBRA-BPS programme might still be considered cost-effective.

To translate these uncertainty results into decision terms for Pakistan, they must be interpreted against a context-appropriate cost-effectiveness threshold. We judge results against an opportunity cost (marginal productivity (MP)) threshold for Pakistan rather than GDP-based thresholds. GDP-based thresholds have been widely criticised as hypothetical and misaligned with real budget trade-offs,^9^ whereas MP thresholds approximate the health that could be generated elsewhere in the system if resources were allocated to alternative services. For Pakistan, the MP-consistent value we apply (US$183 per DALY averted) is substantially lower than GDP-based multiples and therefore more conservative for adoption decisions. In this context, COBRA-BPS adds US$10 per participant in addition to the US$201 per patient-year for routine hypertension care in our model.

Pakistan’s recent Essential Package of Health Services (EPHS) development followed an evidence-informed deliberative process in which cost-effectiveness evidence was considered together with affordability and burden of disease.^30^ This context supports judging COBRA-BPS against an opportunity-cost threshold and considering its cost-effectiveness and budget impact relative to competing EPHS services (eg, immunisation, maternal-newborn care, tuberculosis programmes and primary healthcare-based non-communicable disease (NCD) management), rather than relying on GDP-based thresholds. The programme costs of US$10 per participant reflect programme delivery only (CHW education, blood pressure monitoring/referral, training/supervision, care coordination and administration). This excludes routine healthcare use associated with hypertension of US$201 per patient-year (antihypertensive medications, clinic visits/follow-up and monitoring; acute event costs modelled separately). In our model, although COBRA-BPS adds only US$10 upfront, improved survival and retention in care mean patients accrue more years of the US$201 annual cost, which explains lifetime incremental costs of US$105 per person even as event-related costs decline.

Implementation of new NCD programmes in Pakistan remains debated due to fiscal constraints and competing priorities. Evidence now focuses on how to deliver COBRA-BPS at scale: a Pakistan hybrid type III implementation-effectiveness study is developing and testing strategies to adapt the multicomponent hypertension model to current primary care, including workforce training/supervision, referral and coordination, and supply-chain support for blood pressure control.^29^ Early qualitative work reports high acceptability among clinicians, CHWs and patients and highlights workload pressures for CHWs and medicine availability as recurrent bottlenecks, underscoring that cost-effectiveness is necessary but not sufficient for adoption. In this context, implementation choices (financing of CHW time and supervision, procurement and distribution of blood pressure devices and antihypertensives, and integration with provincial health information systems) are likely to determine whether COBRA-BPS can be delivered at coverage levels that realise its projected value, more than the choice of threshold per se.

## Conclusion

COBRA-BPS delivers meaningful health gains (lower DALYs) but is more costly than standard care and does not appear to be cost-effective at Pakistan’s opportunity cost threshold (US$183 per DALY averted). Policy decisions should therefore prioritise strategies that improve its value for money, for example, integrating COBRA-BPS with existing public health services, pursuing delivery efficiencies, targeting high-risk groups or exploring external/domestic co-financing. While our base case adopts the healthcare (public payer) perspective, the inclusion of wider costs, such as out-of-pocket, non-medical or productivity costs, could potentially influence the conclusions, although the direction and magnitude of this impact remain uncertain.^31^ Further work on implementation efficiencies and country-specific cost data would strengthen policy guidance.

## Supplementary material

10.1136/bmjph-2025-002981 online supplemental file 1

10.1136/bmjph-2025-002981 online supplemental figure 1

## Data Availability

Data sharing is not applicable as no datasets were generated and/or analysed for this study.
