# Supplementary material for: Evaluating the long-term cost-effectiveness of the COBRA-BPS programme in Pakistan
Source: BMJ Public Health. 2025 Dec 3;3(2):e002981. doi: 10.1136/bmjph-2025-002981 (PMC12684083; doi:10.1136/bmjph-2025-002981)
Supplement: online supplemental file 1 [file bmjph-3-2-s001.docx]

# Supplementary Material

**Figure A1. Markov model structure with tunnel states.**

Note: The arrows in this figure represent possible transitions in each cycle. The associated transition probabilities are show in Table A2

This Markov model represents the progression of hypertension and its associated cardiovascular complications over time, capturing the transitions between different health states and their long-term outcomes. The model includes eight health states: Hypertension (the starting state), Myocardial Infarction (MI) and Post MI, Stroke and Post Stroke, Heart Failure (HF) and Post HF, and Death (the absorbing state). The red arrows indicate direct transitions from hypertension to severe outcomes such as MI, stroke, HF, or death, while the green arrows represent disease progression, such as MI leading to heart failure or death. The blue arrows depict chronic disease management states where patients transition from acute events (e.g., MI, stroke, HF) to post-event conditions, while the purple arrows show increased mortality risk following events like stroke. Additionally, loop arrows indicate patients who remain in the same state over multiple cycles. This model is designed to estimate long-term health outcomes, including mortality risk, disease burden (measured in DALYs or life years), and economic costs associated with different disease states. It serves as a framework for evaluating the cost-effectiveness of interventions aimed at preventing or managing hypertension and its complications, providing valuable insights into healthcare decision-making and policy planning.

***Table A1. Baseline covariates for the risk equations***

| *Data Input* | *Base value (SD)* | *Reference* |
| --- | --- | --- |
| *Clinical inputs* |  |  |
| Age | 56.8 (11.5) | [1] |
| Sex (female) % | 61% | [1] |
| SBP | 148.9 (21) | [1] |
| DBP | 91.4 (14.1) | [1] |
| Total Cholesterol | 175 (44.89) | [1] |
| HDL | 42.7 (12.7) | [1] |
| Diastolic blood pressure | 91.4 (14.1) | [1] |
| Systolic Blood pressure | 148.9 (21.0) | [1] |
| Diabetes n (%) | 157 (17.6) | [1] |
| Smoker n (%) | 115 (12.9) | [1] |

**Note:** The data were derived from the baseline characteristics of participants enrolled in the COBRA-BPS trial, which targeted hypertension management in rural communities across Bangladesh, Pakistan, and Sri Lanka. We extracted the values only from Pakistan.

**Table A2. Model parameters**

| ***Variable*** | **Base value** | **Confidence Interval=CI / Credible Interval =CrI** | **Distribution** | **Reference** |
| --- | --- | --- | --- | --- |
| Men (SoC) |  |  |  |  |
| MI | 0.02323 | CrI: (0.0139 - 0.0365) | Multivariate-Normal | Own estimation based on [2] |
| Stroke | 0.0061 | CrI: (0.0029 - 0.0113) | Multivariate-Normal | Own estimation based on [3] |
| HF | 0.00134 | CrI: (0.0007 - 0.0021) | Multivariate-Normal | Own estimation based on [4] |
| Women (SoC) |  |  |  |  |
| MI | 0.02323 | CrI: (0.0139 - 0.0365) | Multivariate-Normal | Own estimation based on [2] |
| Stroke | 0.00369 | CrI: (0.0013 - 0.0083) | Multivariate-Normal | Own estimation based on [3] |
| HF | 0.00018 | CrI: (0.00010 - 0.00032) | Multivariate-Normal | Own estimation based on [4] |
| Men (COBRA-BPS) |  |  |  |  |
| MI | 0.02220 | CrI: (0.0133 - 0.0348) | Multivariate-Normal | Own estimation based on [2] |
| Stroke | 0.0057 | CrI: (0.0028 - 0.0105) | Multivariate-Normal | Own estimation based on [3] |
| HF | 0.00128 | CrI: (0.0007 - 0.00208) | Multivariate-Normal | Own estimation based on [4] |
| Women (COBRA-BPS) |  |  |  |  |
| MI | 0.02220 | CrI: (0.0133 - 0.0348) | Multivariate-Normal | Own estimation based on [2] |
| Stroke | 0.0033 | CrI: (0.00120 - 0.0075) | Multivariate-Normal | Own estimation based on [3] |
| HF | 0.00018 | CrI: (0.00010 - 0.00032) | Multivariate-Normal | Own estimation based on [4] |
| Mortality rate |  |  |  |  |
| All cause male | Age dependent |  | Normal | [5] |
| All cause female | Age dependent |  | Normal | [5] |
| First events |  |  |  |  |
| Stroke | 12.3% for male; 17.8% female | RR 2.23 CI:(1.29 – 3.88) on background mortality | Normal | [6] |
| MI | 5.70% | RR 1.43 CI: (1.18–1.72) on background mortality | Normal | [6] |
| HF | 15.90% | 8% | Normal | [6] |
| Txt effect |  |  |  |  |
| SBP reduction | 4.99 | CI: (0.35 - 9.53) | Normal | [1] |
| Disability weights |  |  |  |  |
| Hypertension | 0.0124 | CI: (0.006 - 0.022) | Beta |  |
| Stroke | 0.07 | CI: (0.046 - 0.099) | Beta | [6] |
| MI | 0.4322 | CI: (0.288 - 0.579) | Beta | [6] |
| HF | 0.0414 | CI: (0.026-0.062) | Beta | [6] |
| Post Stroke | 0.1084 | CI: (0.073 - 0.154) | Beta | [6] |
| Post MI | 0.4322 | CI: (0.288 - 0.579) | Beta | [6] |
| Post HF | 0.1365 | CI: (0.091 - 0.190) | Beta | [6] |

**Note:** Risk equations: Based on Framingham risk equations, which predict cardiovascular risk using clinical risk factors such as age, blood pressure, cholesterol, and diabetes status. Global Burden of Disease (GBD) Study: Age-specific all-cause mortality rates were sourced from the GBD 2021 database. Chen et al.: These risk estimates were derived from observational studies on cardiovascular events following primary and secondary incidents. COBRA-BPS Study: The reduction in systolic blood pressure (SBP) reflects the observed treatment effect of the COBRA-BPS intervention across the trial population.

**Table A3.** Coefficients from the Risk Equation for Myocardial Infarction

| Variable | Men | Women |
| --- | --- | --- |
| Age,-y | 0.04826 | 0.33766 |
| TC,-mg/dL |  |  |
| <160 | -0.65945 | -0.26138 |
| 160-199 | Referent | Referent |
| 200-239 | 0.17692 | 0.20771 |
| 240-279 | 0.50539 | 0.24385 |
| >=280 | 0.65713 | 0.53513 |
| HDL-C,-mg/dL |  |  |
| <35 | 0.49744 | 0.84312 |
| 35-44 | 0.2431 | 0.37796 |
| 45-49 | Referent | 0.19785 |
| 50-59 | -0.05107 | Referent |
| >=60 | -0.4866 | -0.42951 |
| Blood-pressure |  |  |
| Optimal | -0.00226 | -0.53363 |
| Normal | Referent | Referent |
| High-normal | 0.2832 | -0.06773 |
| Stage-I-hypertension | 0.52168 | 0.26288 |
| Stage-II-IV-hypertension | 0.61859 | 0.46573 |
| Diabetes | 0.42839 | 0.59626 |
| Smoker | 0.52337 | 0.29246 |
| Baseline-survival-function-at-10-years, S0(10) | 0.90015 | 0.96246 |
| Linear predictor at risk factor means | 3.0975 | 9.92545 |

**Note:** A standard error equal to 10% of the coefficient was assumed, as the original source did not report standard errors.

**Table A4.** Coefficients from the Risk Equation for Heart Failure.

| **Variable** | **Coefficient** | **SE** |
| --- | --- | --- |
| **Men** | | |
| Intercept | -9.2087 | — |
| Age (10 y) | 0.0412 | 0.0725 |
| LVH (yes/no) | 0.9426 | 0.3485 |
| Heart rate (10 bpm) | 0.0166 | 0.045 |
| SBP (20 mm Hg) | 0.0804 | 0.0583 |
| CHD | 1.6079 | 0.1397 |
| Valve disease | 0.9714 | 0.3221 |
| Diabetes | 0.2244 | 0.1735 |
| **Women** | | |
| Intercept | -10.7988 | — |
| Age (10 y) | 0.0383 | 0.0775 |
| LVH (yes/no) | 1.0933 | 0.3508 |
| Heart rate (10 bpm) | 0.031 | 0.0499 |
| SBP (20 mm Hg) | 0.0857 | 0.0577 |
| CHD | 1.5549 | 0.1546 |
| Valve disease | 1.3857 | 0.2992 |
| Diabetes | 0.1827 | 0.1885 |

**Table A5.** Coefficients from the Risk Equation for Stroke.

| **Risk Factor** | **Men** | **Women** |
| --- | --- | --- |
| Age (per 10 y) | 0.0505 | 0.0657 |
| Systolic blood pressure (per 10 mmHg) | 0.014 | 0.0197 |
| Antihypertensive therapy (yes/no) | 0.3263 | 2.5432 |
| Blood pressure × therapy interaction | 0 | -0.0134 |
| Diabetes mellitus | 0.3384 | 0.5442 |
| Cigarette smoking | 0.5147 | 0.5294 |
| Cardiovascular disease | 0.5195 | 0.4326 |
| Atrial fibrillation | 0.6061 | 1.1497 |
| Left ventricular hypertrophy (LVH) | 0.8415 | 0.8488 |

**Note:** A standard error equal to 10% of the coefficient was assumed, as the original source did not report standard errors.

**Table A6. Cost Estimation**

| ***Category*** | ***Annual cost*** | ***Year*** | ***Base year*** | ***Inflated Cost*** | ***Distribution*** | ***References*** |
| --- | --- | --- | --- | --- | --- | --- |
| Cost per participant | $10.25 | 2016 | 2023 | $ 13.28 | Gamma | [7] |
| Hypertension | $ 201.21 | 2014 | 2023 | $ 263.00 | Gamma | [8] |
| Stroke | $ 1,179.00 | 2015 | 2023 | $ 1,543.00 | Gamma | [9] |
| MI | $ 4,808.00 | 2015 | 2023 | $ 6,295.00 | Gamma | [10] |
| HF | $ 2,435.00 | 2017 | 2023 | $ 3,078.00 | Gamma | [10] |
| Post Stroke | $ 461.81 | 2017 | 2023 | $ 603.00 | Gamma | Own estimation based on [6] |
| Post MI | $ 381.27 | 2017 | 2023 | $ 489.00 | Gamma | Own estimation based on [6] |
| Post HF | $ 568.09 | 2017 | 2023 | $ 706.00 | Gamma | Own estimation based on [6] |

**Note:** *COBRA-BPS Study:* Cost per participant reflects the annual intervention cost, including community health worker engagement, training, and antihypertensive medications. *Hypertension:* Original cost derived from published studies on hypertension management in Pakistan and adjusted to 2023 using the Consumer Price Index (CPI). *Stroke and MI:* Costs were sourced from peer-reviewed studies on cardiovascular disease management in Pakistan and adjusted for inflation. *Heart Failure:* Based on published data from Pakistan-specific health economics studies, reflecting direct medical expenses for inpatient and outpatient care.

**Table A7. Cheers checklist**

| **Section** | **Topic** | **Item** | **Location** |
| --- | --- | --- | --- |
| Title | Title | Identify the study as an economic evaluation and specify the interventions being compared. | page 1 |
| Abstract | Abstract | Provide a structured summary that highlights context, key methods, results, and alternative analyses. | page 2 |
| Introduction | Background and objectives | Give the context for the study, the study question, and its practical relevance for decision making in policy or practice. | page 3 |
| Methods | Health economic analysis plan | Indicate whether a health economic analysis plan was developed and where available. | not reported |
| Methods | Study population | Describe characteristics of the study population (such as age range, demographics, socioeconomic, or clinical characteristics). | page 6 |
| Methods | Setting and location | Provide relevant contextual information that may influence findings. | page 4 |
| Methods | Comparators | Describe the interventions or strategies being compared and why chosen. | page 3 |
| Methods | Perspective | State the perspective(s) adopted by the study and why chosen. | page 6 |
| Methods | Time horizon | State the time horizon for the study and why appropriate. | page 5 |
| Methods | Discount rate | Report the discount rate(s) and reason chosen. | page 7 |
| Methods | Selection of outcomes | Describe what outcomes were used as the measure(s) of benefit(s) and harm(s). | page 7 |
| Methods | Measurement of outcomes | Describe how outcomes used to capture benefit(s) and harm(s) were measured. | page 7 |
| Methods | Valuation of outcomes | Describe the population and methods used to measure and value outcomes. | page 7 |
| Methods | Measurement and valuation of resources and costs | Describe how costs were valued. | page 6 |
| Methods | Currency, price date, and conversion | Report the dates of the estimated resource quantities and unit costs, plus the currency and year of conversion. | page 6 and supplementary material |
| Methods | Rationale and description of model | If modelling is used, describe in detail and why used. Report if the model is publicly available and where it can be accessed. | page 5 |
| Methods | Analytics and assumptions | Describe any methods for analysing or statistically transforming data, any extrapolation methods, and approaches for validating any model used. | pages 5–6 |
| Methods | Characterising heterogeneity | Describe any methods used for estimating how the results of the study vary for subgroups. | page 6 |
| Methods | Characterising distributional effects | Describe how impacts are distributed across different individuals or adjustments made to reflect priority populations. | not reported |
| Methods | Characterising uncertainty | Describe methods to characterise any sources of uncertainty in the analysis. | page 7 |
| Methods | Approach to engagement with patients and others affected by the study | Describe any approaches to engage patients or service recipients, the general public, communities, or stakeholders (such as clinicians or payers) in the design of the study. | not reported |
| Results | Study parameters | Report all analytic inputs (such as values, ranges, references) including uncertainty or distributional assumptions. | supplementary material |
| Results | Summary of main results | Report the mean values for the main categories of costs and outcomes of interest and summarise them in the most appropriate overall measure. | page 8 |
| Results | Effect of uncertainty | Describe how uncertainty about analytic judgments, inputs, or projections affect findings. Report the effect of choice of discount rate and time horizon, if applicable. | page 11 |
| Results | Effect of engagement with patients and others affected by the study | Report on any difference patient/service recipient, general public, community, or stakeholder involvement made to the approach or findings of the study | not reported |
| Discussion | Study findings, limitations, generalisability, and current knowledge | Report key findings, limitations, ethical or equity considerations not captured, and how these could affect patients, policy, or practice. | page 12 |
| Other relevant information | Source of funding | Describe how the study was funded and any role of the funder in the identification, design, conduct, and reporting of the analysis. | page 14 |
| Other relevant information | Conflicts of interest | Report authors conflicts of interest according to journal or International Committee of Medical Journal Editors requirements. | page 14 |

## **References**

1. Jafar, T. H., Gandhi, M., De Silva, H. A., Jehan, I., Naheed, A., Finkelstein, E. A., ... & Feng, L. (2020). A community-based intervention for managing hypertension in rural South Asia. *New England Journal of Medicine*, *382*(8), 717-726.
2. Wilson PW, D'Agostino RB, Levy D, Belanger AM, Silbershatz H, Kannel WB. Prediction of coronary heart disease using risk factor categories. Circulation. 1998 May 12;97(18):1837-47. doi: 10.1161/01.cir.97.18.1837. PMID: 9603539.
3. D'Agostino RB, Wolf PA, Belanger AJ, Kannel WB. Stroke risk profile: adjustment for antihypertensive medication. The Framingham Study. Stroke. 1994 Jan;25(1):40-3. doi: 10.1161/01.str.25.1.40. PMID: 8266381.
4. Kannel WB, D'Agostino RB, Silbershatz H, Belanger AJ, Wilson PW, Levy D. Profile for estimating risk of heart failure. Arch Intern Med. 1999 Jun 14;159(11):1197-204. doi: 10.1001/archinte.159.11.1197. PMID: 10371227.
5. Global Burden of Disease Collaborative Network. *Global Burden of Disease Study 2019 (GBD 2019) Life Tables 1950–2019*. Seattle, WA: Institute for Health Metrics and Evaluation (IHME); 2020.
6. Chen, T., Yu, D., Cornelius, V., Qin, R., Cai, Y., Jiang, Z., & Zhao, Z. (2017). Potential health impact and cost-effectiveness of drug therapy for prehypertension. *International Journal of Cardiology*, *240*, 403-408.
7. Finkelstein, E. A., Krishnan, A., Naheed, A., Jehan, I., de Silva, H. A., Gandhi, M., ... & Tan, S. B. (2021). Budget impact and cost-effectiveness analyses of the COBRA-BPS multicomponent hypertension management programme in rural communities in Bangladesh, Pakistan, and Sri Lanka. *The Lancet Global Health*, *9*(5), e660-e667.
8. Iqbal, M. S., Iqbal, M. W., Bahari, M. B. B., & Iqbal, M. Z. (2014). The Economic Impact Of Hypertension In Health Care System Of Pakistan. *Value in Health*, *17*(7), A485.
9. Khealani, B. A., Javed, Z. F., Syed, N. A., Shafqat, S., & Wasay, M. (2003). Cost of acute stroke care at a tertiary care hospital in Karachi, Pakistan. *Journal of Pakistan Medical Association*, *53*(11), 552.
10. Kumar, A., Siddharth, V., Singh, S. I., & Narang, R. (2022). Cost analysis of treating cardiovascular diseases in a super-specialty hospital. *Plos one*, *17*(1), e0262190.
