# Supplementary figures and images for: Evaluating the long-term cost-effectiveness of the COBRA-BPS programme in Pakistan
Source: BMJ Public Health. 2025 Dec 3;3(2):e002981. doi: 10.1136/bmjph-2025-002981 (PMC12684083; doi:10.1136/bmjph-2025-002981)

Figure A1. Markov model structure

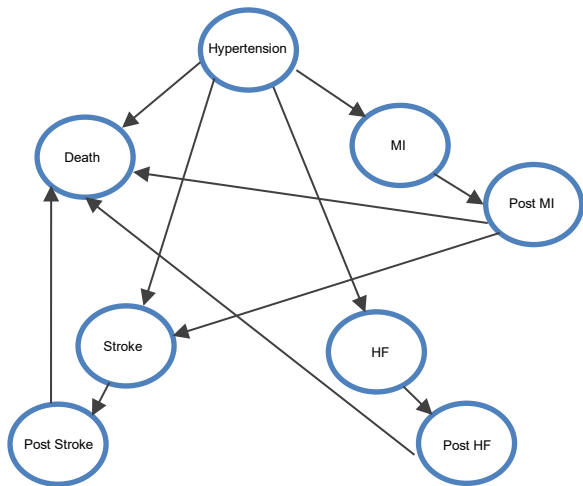

Supplement: online supplemental figure 1 [file bmjph-3-2-s002.pdf]
